# Tissue microarrays: one size does not fit all

**DOI:** 10.1186/1746-1596-5-48

**Published:** 2010-07-07

**Authors:** Jeanette E Eckel-Passow, Christine M Lohse, Yuri Sheinin, Paul L Crispen, Christopher J Krco, Eugene D Kwon

**Affiliations:** 1 Department of Health Sciences Research, Mayo Clinic College of Medicine, Rochester, Minnesota, USA; 2 Department of Pathology and Microbiology, University of Nebraska Medical Center, Omaha, Nebraska, USA; 3 Division of Urology, University of Kentucky, Lexington, Kentucky, USA; 4 Departments of Immunology and Urology, Mayo Clinic College of Medicine, Rochester, Minnesota, USA

## Abstract

**Background:**

Although tissue microarrays (TMAs) are commonly employed in clinical and basic-science research, there are no guidelines for evaluating the appropriateness of a TMA for a given biomarker and tumor type. Furthermore, TMA performance across multiple biomarkers has not been systematically explored.

**Methods:**

A simulated TMA with between 1 and 10 cores was designed to study tumor expression of 6 biomarkers with varied expression patterns (B7-H1, B7-H3, survivin, Ki-67, CAIX, and IMP3) using 100 patients with clear cell renal cell carcinoma (RCC). We evaluated agreement between whole tissue section and TMA immunohistochemical biomarker quantification to assess how many TMA cores are necessary to adequately represent RCC whole tissue section expression. Additionally, we evaluated associations of whole tissue section and TMA expression with RCC-specific death.

**Results:**

The number of simulated TMA cores necessary to adequately represent whole tissue section quantification is biomarker specific. Although 2-3 cores appeared adequate for B7-H3, Ki-67, CAIX, and IMP3, even as many as 10 cores resulted in poor agreement for B7-H1 and survivin compared to RCC whole tissue sections. While whole tissue section B7-H1 was significantly associated with RCC-specific death, no significant associations were detected using as many as 10 TMA cores, suggesting that TMAs can result in false-negative findings if the TMA is not optimally designed.

**Conclusions:**

Prior to TMA analysis, the number of TMA cores necessary to accurately represent biomarker expression on whole tissue sections should be established as there is not a one-size-fits-all TMA. We illustrate the use of a simulated TMA as a cost-effective tool for this purpose.

## Background

Over the last decade, tissue microarrays (TMAs) have become a commonly-used research tool to evaluate associations between biomarkers and clinicopathologic tumor features, patient outcomes and treatment responses. In fact, TMAs are routinely prepared by lead national cancer cooperative groups (e.g., RTOG, SWOG, ECOG, etc.) with the expectation of revealing or testing various biomarkers for prognostication of disease outcome or response to therapy. The appeal of TMAs has been their ability to interrogate hundreds of tissue specimens using a uniform experimental process, while simultaneously preserving limited tissue resources. Although TMAs are convenient and relatively inexpensive, eagerness to exploit this technology has outpaced a comprehensive understanding of its capabilities and limitations. There is no standardized approach to TMA creation, usage or interpretation, particularly for use with multiple biomarkers.

TMAs were initially developed as a high-throughput tool to validate results obtained from gene-expression microarrays [1]. Gene-expression microarray studies are typically performed using only a small number of specimens and thus the identified biomarkers must be validated on hundreds of specimens to evaluate the diagnostic or prognostic value of the candidate biomarker. Even though TMAs were developed to aid this validation process by evaluating hundreds of specimens in a high-throughput manner, Kononen et al. [1] recognized the limitations of sampling only fractions of whole tissue specimens and acknowledged that the results from TMAs may need to be verified by analyzing larger tissue specimens before clinical application.

One of the most recognized limitations of this technology is that the small cores used to construct a TMA may not accurately represent characteristics of the whole tissue specimen [2,3]. To construct a TMA, representative areas from paraffin-embedded, formalin-fixed tumor tissue blocks are selected, cores 0.6-2.0 mm in diameter are punched from the blocks, and the punched samples are subsequently arrayed into a recipient block (referred to as a TMA; see Figure 1 in Giltnane and Rimm 2004 for an in-depth description). Anywhere from 2-4 cores taken from the center, randomly, or peripherally from one or more formalin-fixed tumor tissue blocks are used to create a TMA. Thus, the total area of tumor represented by a TMA can differ by more than 20-fold, ranging from 0.565 to 12.57 mm^2^.

**Figure 1:**
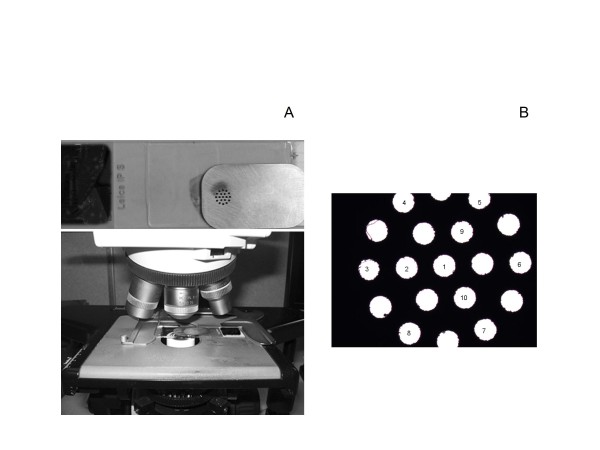
**Simulated TMA template mask**. (A) 19 equally spaced 0.6 mm viewing ports and the TMA template placed below the objective of a microscope, and (B) the order of the 10 cores used for quantification of tumor biomarker expression.

Although it is frequently reported that a TMA has been validated, use of this term is variable. The claim that a TMA has been validated often denotes that the TMA was previously used to find an association between a single biomarker and a single clinicopathologic feature. Details pertaining to how the TMA was validated - for instance, how well the biomarker was represented by the TMA compared to whole tissue sections - are sometimes not published, not well described, or are limited in their interpretation by insufficient sample sizes. There is also a tendency to assume that a TMA can be used to study multiple biomarkers within the same cancer without a thorough understanding of the potentially disparate expression patterns of these biomarkers.

To better understand the consequences of tumor heterogeneity on TMA performance, numerous investigations have compared agreement between TMA and whole tissue section quantification. Based on these studies, a general consensus is that 3-to-4 cores are sufficient to adequately represent whole tissue specimens [4-7]. Camp et al. [8] suggested that 2 cores provide valuable and accurate results, preferably 1 from the tumor edge and 1 from the tumor center. Unfortunately, most studies pertaining to TMA performance have been limited in both size and scope, and have not thoroughly addressed the fundamental issue as to whether a limited number of TMA cores are sufficient across a variety of biomarkers.

Herein, we demonstrate that the design of a TMA is biomarker-specific and that, in fact, a TMA may not be an appropriate tool for some biomarkers. Specifically, the objectives of this study were: (i) to evaluate the agreement between whole tissue section quantification and TMA quantification to assess how many TMA cores are necessary to adequately represent whole tissue section expression for biomarkers with different expression patterns, (ii) to evaluate the suitability of TMAs to identify known associations between biomarker expression and RCC-specific death across biomarkers with different expression patterns, and (iii) to establish the use of a simulated TMA as a cost-effective tool for evaluating the effectiveness of a TMA for a particular study or tumor type. To do so, we simulate the creation of a TMA with as few as 1 and as many as 10 cores to study tumor expression of B7-H1, B7-H3, survivin, Ki-67, CAIX, and IMP3 in clear cell renal cell carcinoma (ccRCC). Although a comparison of TMA and whole tissue sections has been done for survivin [9,10], Ki-67 [11-13], and CAIX [14,15] these comparisons have not been conducted using ccRCC tumors. Furthermore, comparisons of TMA quantification with whole tissue sections for B7-H1, B7-H3 and IMP3 are lacking altogether.

## Methods

### Patient Selection and Outcome

Using the Mayo Clinic Nephrectomy Registry, 100 patients were randomly selected from 351 who were treated with radical or partial nephrectomy for unilateral, sporadic, non-cystic ccRCC between 1995 and 1999. Vital status for patients in the Mayo Clinic Nephrectomy Registry is updated each year. If a patient has died in the previous year, a death certificate is ordered to determine the cause of death. A recent visit to Mayo (within 6 months of the date of death) for metastatic disease is good documentation that RCC was the cause of death. If the death certificate does not support this, the medical history is reviewed by a Mayo urologist to determine the cause of death. If a death certificate cannot be obtained, the cause of death must be verified with the patient's family or local physician.

### Immunohistochemical Staining and Quantification

Sections from paraffin-embedded tissue blocks were immunohistochemically stained for B7-H1, B7-H3, survivin, Ki-67, CAIX, and IMP3 as previously described [16-21]. The membranous and cytoplasmic staining patterns of B7-H1, B7-H3, CAIX, and IMP3 were quantified as the percentage of positive tumor cells in 5-10% increments, whereas the nuclear staining patterns of survivin and KI-67 were quantified as the number of positive tumor cells per mm^2^. Quantification for the original whole tissue sections was conducted by a single pathologist (Y.S.) between August and December of 2006. To provide a measure of intra-reader variability, the study pathologist repeated the whole tissue section quantification for all six biomarkers, blinded to the original whole tissue section quantification. The repeated whole tissue section quantification for the 100 patients was conducted approximately one year after the initial review, between November of 2007 and January of 2008.

### Simulated TMA Construction and Quantification

A simulated TMA was constructed using a stainless steel template mask with dimensions 31 × 20 × 0.15 mm. A Yag laser was used to bore 19 equally-spaced 0.6 mm viewing ports (from core center to core center) in a circular pattern spanning 5 mm in diameter (Figure 1A). The resulting TMA template was thin enough to be placed on a glass slide below the objective of a microscope, yet sturdy enough to be used repeatedly on hundreds of slides. Of the 19 cores on the simulated TMA, only 10 were utilized for manual quantification; the 10 cores were selected to provide representative assessments of both the center of the tumor tissue and the surrounding edges (Figure 1B).

The whole tissue sections that were previously stained for B7-H1, B7-H3, survivin, KI-67, CAIX, and IMP3 were collected and the patient information on each slide was replaced with study identifiers ranging from 1 to 100 for each biomarker. To blind the pathologist, only the technicians and statisticians had access to the link between the patient information and the identifiers. For TMA quantification, the pathologist first viewed each slide under low magnification to determine the center of the tumor tissue, without regard to the immunohistochemical staining pattern. The center of the TMA template was then placed on the area of the slide that contained the center of the tumor tissue. Subsequently, tumor biomarker expression was quantified for the 10 labeled cores on the TMA template for each slide (Figure 1B). For cores that contained at least 25% tumor tissue, the membranous and cytoplasmic staining patterns of B7-H1, B7-H3, CAIX, and IMP3 were quantified as the percentage of positive tumor cells in 5-10% increments, whereas the nuclear staining patterns of survivin and KI-67 were quantified as the number of positive tumor cells per mm^2^. Cores that contained primarily normal, stromal, artifact, necrotic, or degenerative tissue and cores that did not contain any tissue at all were recorded as such.

### Statistical Methods

The goals of this study were: (i) to evaluate the agreement between whole tissue section quantification and TMA quantification to assess how many TMA cores are necessary to adequately represent whole tissue section expression for biomarkers with different expression patterns, and (ii) to evaluate the suitability of TMAs to identify known associations between biomarker expression and RCC-specific death across biomarkers with different expression patterns.

To evaluate the agreement between whole tissue section quantification and TMA quantification, the agreement between repeated whole tissue section quantification (i.e., whole tissue section intra-reader agreement) was first evaluated as a point of reference. Note that the first and second whole tissue section quantifications were performed approximately one year apart. Whole tissue section intra-reader agreement was measured using the kappa statistic [22] and the concordance correlation coefficient (CCC) [23]. The kappa statistic is designed for categorical data and is a measure of the difference between the observed agreement and the amount of agreement that would be expected by chance. Kappa statistics of 0.01-0.20, 0.21-0.40, 0.41-0.60, 0.61-0.80, and 0.81-0.99 represent slight, fair, moderate, substantial, and almost perfect agreement, respectively. The CCC is designed for continuous data and measures the variation from the 45-degree line through the origin (the concordance line); values range from 0.0 to 1.0, with higher values indicating a greater level of agreement [23].

Since the kappa statistics and CCCs indicated that the whole tissue section intra-reader agreement was good, we compared the simulated TMA quantification with the second whole tissue section quantification. The goal was to evaluate how many cores (from 1 and up to 10) are needed to achieve adequate agreement in biomarker expression between the simulated TMA and the whole tissue section. Bootstrap resampling was performed to simulate varying numbers of cores. For each bootstrap sample, the simulated number of cores (from 1 and up to 10) was randomly sampled from each patient without replacement and the expression values across the corresponding informative cores for each patient were summarized by the maximum expression value for B7-H1, B7-H3, survivin, KI-67, and IMP3 and by the minimum expression value for CAIX. Note that higher expressions of B7-H1, B7-H3, survivin, KI-67, and IMP3 are associated with RCC-specific death, whereas lower expression of CAIX is associated with RCC-specific death [16-21]. The bootstrap procedure was executed 500 times for each simulated number of cores. Agreement between the summarized TMA quantification and the second whole tissue section quantification was evaluated for all patients with at least one informative core using the kappa statistic and the CCC. The TMA template was designed to simulate the creation of TMAs with as few as 1 and as many as 10 cores, some of which could be non-informative due to heterogeneity in tumor tissue or the processing of the TMA template. As such, the bootstrap resampling procedure could choose a non-informative core, which in fact mimics a real TMA.

To evaluate associations of whole tissue section and TMA biomarker expression with RCC-specific death, bootstrap resampling was used to evaluate associations of biomarker expression with RCC-specific death using 1 and up to 10 cores [7]. For each bootstrap sample, the simulated number of cores (from 1 and up to 10) was randomly sampled from each patient without replacement and the expression values across the corresponding cores for each patient were summarized by the maximum expression value for B7-H1, B7-H3, survivin, KI-67, and IMP3 and by the minimum expression value for CAIX. The bootstrap procedure was executed 500 times for each simulated number of cores. Cox proportional hazards regression was used to evaluate the association between biomarker expression and RCC-specific death via the Wald chi-square statistic. The duration of follow-up was calculated from the date of surgery to the date of death or last follow-up as of February 2008. Patients who died from causes other than RCC were censored at the date of death; patients who were still alive at the time of analysis were censored at the date of last follow-up.

## Results and Discussion

The clinicopathologic features for the 100 randomly selected patients are described in Table 1. Patients were mostly male (67%), ECOG 0 (77%), pNX/pN0 (96%), M0 (96%), and TNM stage group I (56%). These clinicopathologic features are representative of the available 351 ccRCC patients from our 1995 to 1999 cohort (data not shown). At last follow-up, 50 of the 100 patients had died, including 24 who died from RCC at a median of 1.6 years following surgery (range 0.2 - 10.5). Among the 50 patients who were still alive, the median duration of follow-up was 9.1 years (range 2.3 - 13.0).

**Table 1:** Clinicopathologic features for 100 patients treated surgically for ccRCC between 1995 and 1999

Feature	N
Gender	
Female	33
Male	67
Symptomatic at Presentation	51
ECOG Performance Status	
0	77
1	21
2	2
2002 Primary Tumor Classification	
pT1a	25
pT1b	32
pT2	18
pT3a	10
pT3b	13
pT3c	0
pT4	2
Regional Lymph Node Involvement	
pNX/pN0	96
pN1/pN2	4
Distant Metastases	
M0	96
M1	4
2002 TNM Stage Groupings	
I	56
II	16
III	21
IV	7
Nuclear Grade	
1	2
2	57
3	33
4	8
Coagulative Tumor Necrosis	21
Sarcomatoid Differentiation	4

A summary of the whole tissue section quantification for each biomarker is provided in Table 2. B7-H1 expression was nearly dichotomous for the 100 ccRCC tumors studied; there were 92, 7, and 1 tumor with 0%, 5%, and 10% B7-H1 expression, respectively. As such, B7-H1 expression was analyzed as positive (i.e., 5% or greater) versus negative. Before evaluating the agreement in biomarker expression between whole tissue section quantification and TMA quantification, we first evaluated whole tissue section intra-reader agreement. The two whole tissue section readings were conducted by a single pathologist approximately one year apart. The kappa statistic for B7-H1 was 0.78, signifying substantial agreement between the repeated whole tissue section quantifications. The CCCs for survivin, Ki-67, CAIX, IMP3, and B7-H3 were 0.71, 0.90, 0.94, 0.96, and 0.97, respectively (Table 3). As shown in Additional file 1, Figure S1, the intra-reader agreement for survivin and Ki-67 was a function of expression since the magnitude of disagreement increased as expression increased.

**Table 2:** Summary of biomarker expression for the second whole tissue section quantification

		Distribution of expression values						
Biomarker	Quantitation Method^*^	0% (min)	10%	25%	50% (median)	75%	90%	100% (max)
B7-H1	%	0	0	0	0	0	0	10
B7-H3	%	0	0	0	0	0	5	100
IMP3	%	0	0	0	0	0	10	80
CAIX	%	0	20	100	100	100	100	100
Survivin	mm^2^	0.65	1.95	2.61	4.56	12.38	25.41	82.08
Ki-67	mm^2^	0.65	2.61	12.05	24.76	41.69	68.08	329.64

*The membranous and cytoplasmic staining patterns of B7-H1, B7-H3, IMP3, and CAIX were quantitated as the percentage of positive tumor cells in 5-10% increments, whereas the nuclear staining patterns of survivin and KI-67 were quantitated as the number of positive tumor cells per mm^2^.

**Table 3:** Whole tissue section intra-reader agreement and agreement between the second whole tissue section and TMA

	B7-H1	B7-H3	Survivin	Ki-67	CAIX*	IMP3
	Kappa	CCC	CCC	CCC	CCC	CCC
Whole section	0.78	0.97	0.71	0.90	0.94	0.96
Cores						
1	0.21 (0.00 - 0.65)	0.80 (0.73 - 0.87)	0.35 (0.06 - 0.55)	0.63 (0.44 - 0.82)	0.85 (0.73 - 0.94)	0.73 (0.02 - 0.87)
2	0.21 (0.00 - 0.65)	0.81 (0.74 - 0.87)	0.40 (0.12 - 0.57)	0.72 (0.49 - 0.84)	0.82 (0.73 - 0.92)	0.79 (0.20 - 0.85)
3	0.38 (0.00 - 0.65)	0.83 (0.76 - 0.86)	0.43 (0.24 - 0.62)	0.76 (0.62 - 0.86)	0.80 (0.72 - 0.89)	0.81 (0.49 - 0.85)
4	0.38 (0.00 - 0.65)	0.83 (0.77 - 0.85)	0.44 (0.33 - 0.58)	0.79 (0.64 - 0.85)	0.78 (0.71 - 0.87)	0.82 (0.61 - 0.85)
5	0.52 (0.00 - 0.65)	0.83 (0.80 - 0.85)	0.45 (0.36 - 0.57)	0.81 (0.71 - 0.86)	0.77 (0.72 - 0.84)	0.82 (0.77 - 0.85)
6	0.52 (0.00 - 0.65)	0.84 (0.81 - 0.84)	0.45 (0.37 - 0.57)	0.81 (0.71 - 0.85)	0.76 (0.71 - 0.82)	0.82 (0.77 - 0.85)
7	0.65 (0.00 - 0.65)	0.84 (0.81 - 0.84)	0.46 (0.40 - 0.52)	0.82 (0.73 - 0.85)	0.75 (0.72 - 0.82)	0.82 (0.77 - 0.85)
8	0.65 (0.38 - 0.65)	0.84 (0.83 - 0.84)	0.46 (0.41 - 0.51)	0.82 (0.76 - 0.84)	0.74 (0.72 - 0.81)	0.82 (0.79 - 0.85)
9	0.65 (0.52 - 0.65)	0.84 (0.83 - 0.84)	0.46 (0.43 - 0.48)	0.82 (0.79 - 0.84)	0.73 (0.72 - 0.79)	0.82 (0.80 - 0.84)
10	0.65 (0.65 - 0.65)	0.84 (0.84 - 0.84)	0.46 (0.46 - 0.46)	0.82 (0.82 - 0.82)	0.73 (0.73 - 0.73)	0.82 (0.82 - 0.82)

*CAIX was analyzed using the minimum value across cores. All other biomarkers were analyzed using the maximum value across cores.

The kappa statistic is reported for B7-H1, whereas the concordance correlation coefficient (CCC) is reported for B7-H3, survivin, Ki-67, CAIX, and IMP3; the median (minimum, maximum) statistic from the 500 bootstrap samples is provided.

Allowing the whole tissue section intra-reader agreement to serve as a point of reference, we evaluated the agreement in biomarker expression between the second whole tissue section quantification and the quantification using a simulated TMA. Table 3 provides measures of agreement between the summarized TMA and the second whole tissue section quantifications. The minimum, median, and maximum agreement measure (kappa or CCC) from the 500 bootstrap simulations is provided for each biomarker. Using only 2 cores, the median kappa statistic for B7-H1 was 0.21 and the median CCCs for survivin, Ki-67, IMP3, B7-H3, and CAIX were 0.40, 0.72, 0.79, 0.81, and 0.82, respectively. The results for 3 cores were very similar to those of 2 cores. Although 2-to-3 cores might be adequate for Ki-67, IMP3, B7-H3, and CAIX (CCC > 0.70), more than 3 cores are clearly necessary for B7-H1 and survivin. As such, we observed that the number of cores necessary for a TMA to adequately represent whole tissue section quantification is clearly not the same for all biomarkers. Even when using all 10 cores, the agreement between the TMA and whole tissue section was noticeably worse than the intra-reader agreement between repeated quantifications of the whole tissue section.

**Figure 2:**
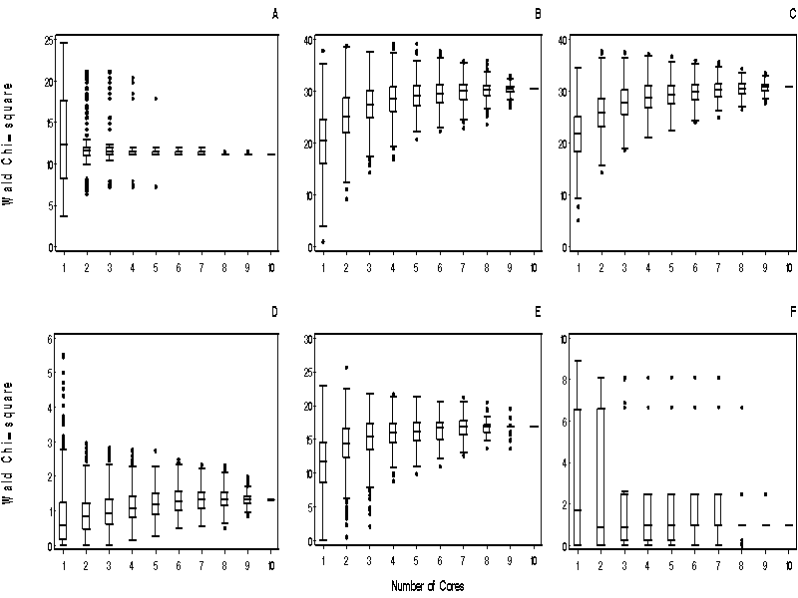
**Box plots illustrating Wald chi-square statistics from 500 bootstrap samples**. Testing associations of (A) B7-H3, (B) survivin, (C) Ki-67, (D) CAIX, (E) IMP3, and (F) B7-H1 expression with RCC-specific death using 1 and up to 10 cores. The lower, middle, and upper horizontal lines that comprise the boxes denote the 25^th ^percentile, median, and 75^th ^percentile of the distribution of test statistics from the 500 bootstrap samples. Using a significance level of 0.05, the critical value for a 1 degree-of-freedom chi-square test statistic is approximately 3.8. B7-H1 was modeled as dichotomous (positive versus negative), whereas B7-H3, survivin, Ki-67, CAIX, and IMP3 were modeled as continuous.

Significant associations between B7-H1, B7-H3, survivin, Ki-67, IMP3, and CAIX with RCC-specific death have been reported in the literature using whole tissue section quantification [16-21] and thus our goal was to determine if a TMA could corroborate these associations. For the Cox analysis, B7-H1 was modeled as dichotomous (positive versus negative), whereas the remaining biomarkers were modeled as continuous. Table 4 provides a summary of associations of the second whole tissue section quantification with RCC-specific death. Figure 2 illustrates the distribution of Wald chi-square statistics from 500 bootstrap samples testing associations of biomarker expression with RCC-specific death using 1 and up to 10 cores from the simulated TMA. Using a significance level of 0.05, the critical value for a 1 degree-of-freedom chi-square test statistic is approximately 3.8. For B7-H3 (Figure 2A), the median chi-square statistic remained stable from 1 to 10 cores; however, the variability associated with the statistics (i.e., the width of the boxes) decreased dramatically from 1 to 2 cores. For survivin, Ki-67, CAIX, and IMP3 (Figures 2B-E) the median chi-square statistic became stable after 4 to 5 cores. Furthermore, the 25^th ^percentile of chi-square values increased as the number of cores increased, suggesting that the association between expression and RCC-specific death was strengthened by using more cores. Even though the associations between CAIX and RCC-specific death (Figure 2D) were strengthened by using more cores, most of the chi-square statistics were less than 3.84 and thus the associations were non-significant regardless of the number of cores studied. This result is not surprising since the association of CAIX with RCC-specific death using whole tissue sections was also non-significant (Table 4), suggesting that our study of 100 patients lacks statistical power to detect associations of CAIX expression with outcome. Lastly, although use of whole tissue sections showed a significant association of B7-H1 expression with RCC-specific death (Table 4), most of the chi-square statistics from the simulated TMA were less than 3.84 (Figure 2F), regardless of the number of cores studied. Therefore, B7-H1 is an example of a focal and rarely expressed biomarker that is not well suited for TMA analysis when evaluating the association of expression with RCC-specific death.

**Table 4:** Associations of whole tissue section quantification with RCC-specific death

Second Whole Section Quantification	**Wald χ**^**2**^	Hazard Ratio (95% CI)	P-value
B7-H1 (positive versus negative)	7.7	4.09 (1.51 - 11.08)	0.006
B7-H3 (increase of 5%)	11.7	1.14 (1.06 - 1.24)	< 0.001
Survivin (increase of 5-cell per mm^2^)	31.3	1.32 (1.20 - 1.46)	< 0.001
Ki-67 (increase of 5-cell per mm^2^)	30.2	1.07 (1.04 - 1.10)	< 0.001
CAIX (decrease of 5%)	0.1	1.01 (0.92 - 1.10)	0.863
IMP3 (increase of 5%)	24.9	1.31 (1.18 - 1.45)	< 0.001

## Conclusions

While it is frequently reported in the literature that a TMA has been previously validated, details pertaining to this validation are often absent or are not well described. In many instances, the observed association of TMA biomarker expression with a single clinicopathologic feature of the cancer of interest is provided as evidence that the TMA has been validated and TMA technology can therefore be used to study other biomarkers in the same cancer or the same biomarker in other cancers. As such, prior to preparing a TMA, the number of TMA cores necessary to accurately represent whole tissue section biomarker expression should be established on a biomarker-specific and cancer-specific basis. We established the use of a simulated TMA as a cost-effective tool for this purpose.

The first objective of this study was to evaluate agreement between whole tissue section quantification and quantification using a simulated TMA to determine the number of TMA cores necessary to adequately represent whole tissue section expression for 6 biomarkers that have been shown to be associated with ccRCC patient outcome [16-21]. The biomarkers studied displayed different frequencies and patterns of expression; consequently, the number of TMA cores necessary to adequately represent whole tissue sections was not the same for all biomarkers. In particular, using a simulated TMA, we determined that 2-to-3 cores appeared adequate for Ki-67, IMP3, B7-H3, and CAIX, but even as many as 10 cores resulted in poor agreement for B7-H1 and survivin with their whole tissue section counterparts. This demonstrates that the number of TMA cores necessary to represent whole tissue specimens is biomarker-specific and thus a single TMA is not always appropriate for a set of markers.

The second objective of this study was to evaluate the ability of a TMA to discover associations between biomarker expression and patient outcome. For most of the biomarkers studied, 2-to-4 cores appeared adequate to identify the associations between expression and outcome observed with whole tissue sections. However, while whole tissue section B7-H1 was significantly associated with RCC-specific death, no significant associations with patient outcome were detected using as many as 10 cores for this focal and rarely expressed biomarker, demonstrating that a TMA may not be an appropriate tool for some biomarkers. The suitability of TMA analysis of B7-H1 for other studies and tumor types need to be further evaluated. It is important to identify biomarkers that are not well suited for TMA analysis in order to eliminate false-negative conclusions obtained from TMA analyses. Likewise, the sample size necessary to detect associations is not equivalent for all biomarkers as a result of the varying expression patterns across biomarkers.

TMAs are commonly used to test various biomarkers for their ability to predict disease outcome or response to therapy since TMAs allow investigators to study many tissue specimens using a uniform experimental process, while simultaneously preserving limited tissue resources [24]. As the results of the current study demonstrate, the general guideline that 3-to-4 cores are sufficient to adequately represent whole tissue specimens does not hold true for all biomarkers, and presumably for all malignancies. Our results corroborate the conclusions of Fons et al. [5] who suggested that the concordance between TMA and whole tissue section quantification depends on the expression pattern of the biomarker. Biomarkers with focal and rare expression may not be well suited for TMA analysis, as Linderoth et al. [25] demonstrated for BCL6 expression in diffuse large B-cell lymphoma, whereas biomarkers with diffuse expression may be adequately represented by a limited number of TMA cores. Although it may be concluded that TMAs are being successfully used since they are uncovering significant associations between biomarker expression and outcome, we highlight the likelihood of obtaining false-negative findings when using a single TMA to quantify multiple biomarkers with heterogeneous patterns of expression.

One limitation of our study is that we did not fully evaluate potential sources of variability in biomarker expression due to non-informative cores and core location. However, as shown in Table 5, we did observe that the outer ring of cores on the simulated TMA were more likely to contain primarily normal, stromal, artifact, necrotic, degenerative tissue or no tissue at all in comparison to the inner ring of cores. The non-informative cores were retained throughout the analyses, reasoning that they represented missing cores on a real TMA (i.e., cores that are lost during processing or that contained minimal tumor). For example, Fons et al. [5] reported that 10%, 8%, and 9% of TMA cores were not assessable for their study of oestrogen and progesterone receptor, p53, and epithelial membrane antigen, respectively. Similarly, Gillett et al. [26] reported that 12% and 13% of TMA cores had floated off during the immunohistochemical technique and 10% and 12% of the stained sections did not contain invasive tumor when evaluating estrogen and progesterone receptors, respectively. Thus, the number of non-informative cores observed with our simulated TMA is similar to what others have observed using real TMAs. Additionally, using the simulated TMA, we were unable to evaluate variability in biomarker expression associated with core size or different slices of the TMA block. With respect to core size, Lesnikova et al. [24] evaluated cervical neoplasia specimens and concluded that 1 mm tissue cores were more appropriate than 0.6 or 2 mm cores and provide a suitable compromise of being large enough to be representative yet small enough to be high throughput. With respect to evaluating different slices of the TMA block, Hager et al. [4] evaluated RCC specimens and concluded that the percentage of lost or non-informative cores tripled from the first slice to the last slice of the TMA block primarily due to the presence of limited (> 25%) tumor tissue, core folding, and necrotic tissue. Lastly, the use of a TMA template mask placed on previously stained whole tissue sections did not allow us to address additional variability that can be introduced by day-to-day fluctuations in staining procedures or TMA core edge effects.

**Table 5:** Number of non-informative cores observed (out of 100 patients) on the simulated TMA

Core	B7-H1	B7-H3	Survivin	Ki-67	CAIX	IMP3
1	5	7	5	4	13	15
2	3	7	8	4	9	16
**3**	**4**	**7**	**7**	**7**	**17**	**20**
**4**	**6**	**6**	**14**	**10**	**21**	**13**
**5**	**5**	**8**	**13**	**6**	**15**	**12**
**6**	**6**	**7**	**9**	**9**	**12**	**15**
**7**	**5**	**5**	**10**	**11**	**28**	**13**
**8**	**4**	**4**	**11**	**7**	**20**	**8**
9	4	2	6	3	7	8
10	3	7	3	3	9	13

Cores 1, 2, 9, and 10 denote the inner ring and cores 3-8 denote the outer ring of the simulated TMA (Figure 1B).

While non-informative cores, core location, core size, repeated sectioning of the TMA block, and staining fluctuations certainly contribute to variability in biomarker expression, variability may also arise by the use of a single TMA to study multiple biomarkers without an understanding of their varied expression patterns in whole tissue sections. This often underestimated variability may contribute to the inability of promising biomarkers to achieve clinical utility. As McShane et al. [27] remarked, while numerous biomarkers have been studied, the number of biomarkers that have attained clinical utility is "pitifully small". Initial reports of a biomarker's promising ability to predict a clinical outcome are rarely substantiated by subsequent studies of the same biomarker or related biomarkers. McShane and colleagues cite a number of reasons for these inconsistent findings including insufficient sample size, inappropriate statistical methods, and use of biomarker assays that are not standardized and reproducible. Herein, we demonstrate that studies that employ TMA technology without first evaluating agreement in biomarker expression with whole tissue sections may further contribute to washout of biomarker utility in the clinical setting.

In summary, TMAs are useful for studying a given biomarker provided that the number of TMA cores necessary to accurately represent whole tissue section biomarker expression is established on a biomarker-specific and tumor-specific basis as there is not a one-size-fits-all TMA. We recommend that guidelines for evaluating the appropriateness of a TMA for a given biomarker and tumor type should be developed, similar to the REMARK guidelines established for prognostic studies [27]. We further recommend that any claims suggesting that a TMA can be used to represent biomarker expression in whole tissue sections be supported by published data that disclose rigorous evidence of these validation steps.

## Abbreviations

CCC: concordance correlation coefficient; ccRCC: clear cell renal cell carcinoma; RCC: renal cell carcinoma; TMA: tissue microarray

## Competing interests

EDK and Mayo Clinic have received royalties greater than the federal threshold for significant financial interest from the licensing to Medarex of technology related to B7-H1 and have contractual rights to receive future royalties from the licensing of this technology. Some authors (C.M.L., Y.S., E.D.K) have filed patents for the potential use of B7-H1, B7-H3, survivin, or Ki-67 as prognostic markers for the assessment of cancer.

## Authors' contributions

JEEP participated in the design and coordination and helped draft the manuscript. CML participated in the design, performed the statistical analyses and helped draft the manuscript. YS participated in the design and read the whole section and TMA slides. PLC and CJK participated in the design and helped draft the manuscript. EDK conceived of they study, participated in the design and coordination and helped draft the manuscript. All authors read and approved the final manuscript.

## Supplementary Material

Additional file 1 **Figure S1: Bland-Altman plots of the whole tissue section intra-reader agreement for (A) survivin and (B) Ki-67**. Click here for file

## References

[B1] Kononen J Bubendorf L Kallioniemi A Bärlund M Schraml P Leighton S Torhorst J Mihatsch MJ Sauter G Kallioniemi OP Tissue microarrays for high-throughput molecular profiling of tumor specimens Nat Med 1998 4 844 847 10.1038/nm0798-844 9662379

[B2] Giltnane JM Rimm DL Technology insight: identification of biomarkers with tissue microarray technology Nat Clin Pract Oncol 2004 1 104 111 10.1038/ncponc0046 16264828

[B3] Simon R Mirlacher M Sauter G Tissue microarrays in cancer diagnosis Expert Rev Mol Diagn 2003 3 421 430 10.1586/14737159.3.4.421 12877382

[B4] Hager M Kolbitsch C Tiefenthaler W Haufe H Kemmerling R Moser PL Tissue microarrays from renal cell tumors: exclusion criteria and rate of exclusion Scand J Urol Nephrol 2007 41 485 489 10.1080/00365590701520552 17853046

[B5] Fons G Hasibuan SM van der Velden J ten Kate FJW Validation of tissue microarray technology in endometrioid cancer of the endometrium J Clin Pathol 2007 60 500 503 10.1136/jcp.2006.040170 16822874 PMC1994552

[B6] Aaltonen K Ahlin C Amini RM Salonen L Fjällskog ML Heikkilä P Nevanlinna H Blomqvist C Reliability of cyclin A assessment on tissue microarrays in breast cancer compared to conventional histological slides Br J Cancer 2006 94 1697 1702 16670718 10.1038/sj.bjc.6603147 PMC2361315

[B7] Rubin MA Dunn R Strawderman M Pienta KJ Tissue microarray sampling strategy for prostate cancer biomarker analysis Am J Surg Pathol 2002 26 312 319 10.1097/00000478-200203000-00004 11859202

[B8] Camp RL Charette LA Rimm DL Validation of tissue microarray technology in breast carcinoma Lab Invest 2000 80 1943 1949 10.1038/labinvest.3780204 11140706

[B9] Lusis EA Chicoine MR Perry A High throughput screening of meningioma biomarkers using a tissue microarray J Neurooncol 2005 73 219 223 10.1007/s11060-004-5233-y 15980972

[B10] Garcia JF Camacho FI Morente M Fraga M Montalbán C Álvaro T Bellas C Castaño Á Diez A Flores T Martin C Martinez M Mazorra F Menárguez J Mestre M Mollejo M Sáez A Sánchez L Piris M Hodgkin and Reed-Sternberg cells harbor alterations in the major tumor suppressor pathways and cell-cycle checkpoints: analyses using tissue microarrays Blood 2003 101 681 689 10.1182/blood-2002-04-1128 12393683

[B11] Cunha KS Caruso AC Goncalves AS Bernardo VG Pires AR da Fonseca EC de Faria PA da Silva LE Geller M de Moura-Neto RS Lopes VS Validation of tissue microarray technology in malignant peripheral nerve sheath tumours J Clin Pathol 2009 62 629 633 10.1136/jcp.2008.063081 19318344

[B12] Hecht JL Kotsopoulos J Gates MA Hankinson SE Tworoger SS Validation of tissue microarray technology in ovarian cancer: results from the Nurses' Health Study Cancer Epidemiol Biomarkers Prev 2008 17 3043 3050 10.1158/1055-9965.EPI-08-0645 18990746 PMC2628443

[B13] Fons G van der Velden J Burger M ten Kate F Validation of tissue microarray technology in vulvar cancer Int J Gynecol Pathol 2008 28 76 82 10.1097/PGP.0b013e3181817b2a 19047904

[B14] Maseide K Pintilie M Kandel R Hill RP Can sparsely and heterogeneously expressed proteins be detected using tissue microarrays? A simulation study of the hypoxia marker carbonic anhydrase IX (CA IX) in human soft tissue sarcoma Pathol Res Pract 2008 204 175 183 10.1016/j.prp.2007.10.009 18180112

[B15] Iakovlev VV Pintilie M Morrison A Fyles AW Hill RP Hedley DW Effect of distributional heterogeneity on the analysis of tumor hypoxia based on carbonic anhydrase IX Lab Invest 2007 87 1206 1217 10.1038/labinvest.3700680 17906661

[B16] Thompson RH Kuntz SM Leibovich BC Dong H Lohse CM Webster WS Sengupta S Frank I Parker AS Zincke H Blute ML Sebo TJ Cheville JC Kwon ED Tumor B7-H1 is associated with poor prognosis in renal cell carcinoma patients with long-term follow-up Cancer Res 2006 66 3381 3385 10.1158/0008-5472.CAN-05-4303 16585157

[B17] Crispen PL Sheinin Y Roth TJ Lohse CM Kuntz SM Frigola X Thompson RH Boorjian SA Dong H Leibovich BC Blute ML Kwon ED Tumor cell and tumor vasculature expression of B7-H3 predict survival in clear cell renal cell carcinoma Clin Cancer Res 2008 14 5150 5157 10.1158/1078-0432.CCR-08-0536 18694993 PMC2789387

[B18] Parker AS Lohse CM Leibovich BC Cheville JC Sheinin YM Kwon ED Comparison of digital image analysis versus visual assessment to assess survivin expression as an independent predictor of survival for patients with clear cell renal cell carcinoma Hum Pathol 2008 39 1176 1184 10.1016/j.humpath.2007.12.009 18538369 PMC2789391

[B19] Tollefson MK Thompson RH Sheinin Y Lohse CM Cheville JC Leibovich BC Kwon ED Ki-67 and coagulative tumor necrosis are independent predictors of poor outcome for patients with clear cell renal cell carcinoma and not surrogates for each other Cancer 2007 110 783 790 10.1002/cncr.22840 17594714

[B20] Leibovich BC Sheinin Y Lohse CM Thompson RH Cheville JC Zavada J Kwon ED Carbonic anhydrase IX is not an independent predictor of outcome for patients with clear cell renal cell carcinoma J Clin Oncol 2007 25 4757 4764 10.1200/JCO.2007.12.1087 17947723

[B21] Hoffmann NE Sheinin Y Lohse CM Parker AS Leibovich BC Jiang Z Kwon ED External validation of IMP3 expression as an independent prognostic marker for metastatic progression and death for patients with clear cell renal cell carcinoma Cancer 2008 112 1471 1479 10.1002/cncr.23296 18260086 PMC2792740

[B22] Landis JR Koch GG The measurement of observer agreement for categorical data Biometrics 1977 33 159 174 10.2307/2529310 843571

[B23] Lin L A concordance correlation coefficient to evaluate reproducibility Biometrics 1989 45 255 268 10.2307/2532051 2720055

[B24] Lesnikova I Lidang M Hamilton-Dutoit S Koch J p16 as a diagnostic marker of cervical neoplasia: a tissue microarray study of 796 archival specimens Diagn Pathol 2009 4 22 10.1186/1746-1596-4-22 19589135 PMC2714065

[B25] Linderoth J Ehinger M Akerman M Cavallin-Ståhl E Enblad G Erlanson M Jerkeman M Tissue microarray is inappropriate for analysis of BCL6 expression in diffuse large B-cell lymphoma Eur J Haematol 2007 79 146 149 10.1111/j.1600-0609.2007.00892.x 17635238

[B26] Gillett CE Springall RJ Barnes DM Hanby AM Multiple tissue core arrays in histopathology research: a validation study J Pathol 2000 192 549 553 10.1002/1096-9896(2000)9999:9999<::AID-PATH721>3.0.CO;2-0 11113875

[B27] McShane L Altman D Sauerbrei W Taube S Gion M Clark G Reporting recommendations for tumor marker prognostic studies (REMARK) J Natl Cancer Inst 2005 97 1180 1184 10.1093/jnci/dji237 16106022

