# Supplementary material for: Tissue microarrays: one size does not fit all
Source: Diagn Pathol. 2010 Jul 7;5:48. doi: 10.1186/1746-1596-5-48 (PMC2910003; doi:10.1186/1746-1596-5-48)
Supplement: Additional file 1 — Figure S1: Bland-Altman plots of the whole tissue section intra-reader agreement for (A) survivin and (B) Ki-67. [file 1746-1596-5-48-S1.PDF]

ADDITIONAL FILE 1

Figure S1: Bland-Altman plots of the whole tissue section intra-reader agreement for (A) Survivin and (B) Ki-67. A Bland-Altman plot [1] is a plot of the difference between two measurements (A – B) against the average of the two measurements (A + B) / 2. In comparison to a simple correlation plot of A versus B, a Bland-Altman plot provides a better visualization of the magnitude of disagreement (error and bias) and better highlights outliers and trends in the disagreement. If the differences between two measurements are not related to the magnitude of either measurement, then it is expected that the data will be randomly scattered around the zero horizontal reference line. These plots revealed that for survivin and Ki-67 the intra-reader agreement was a function of expression since the magnitude of disagreement increased as expression increased.

REFERENCES:

1. Altman DG, Bland JM: **Measurement in medicine: the analysis of method comparison studies.** *The Statistician* 1983, **32:** 307-317.

(A)

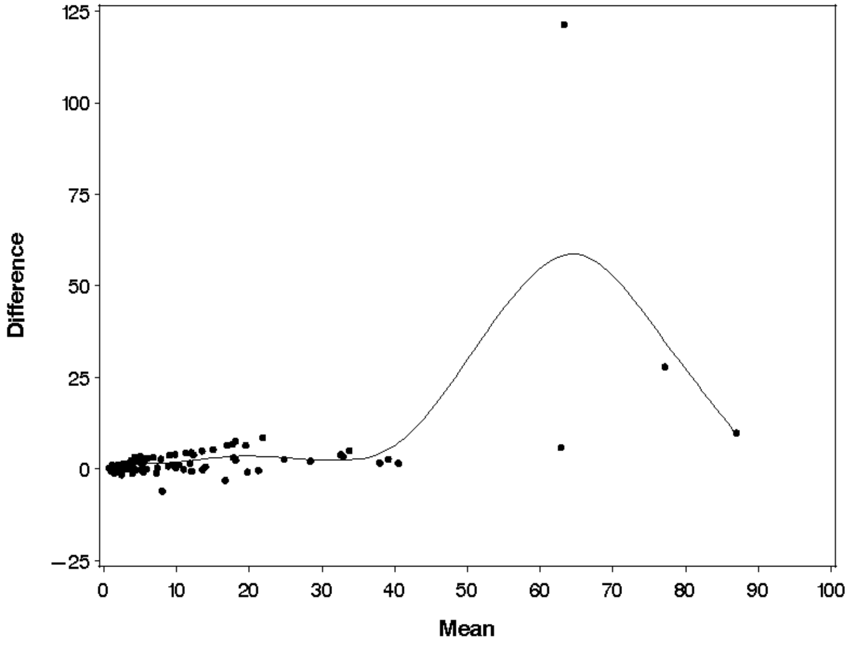
125
100
75
50
25
0
−25
Difference
0
10
20
30
40
50
60
70
80
90
100
Mean

(B)

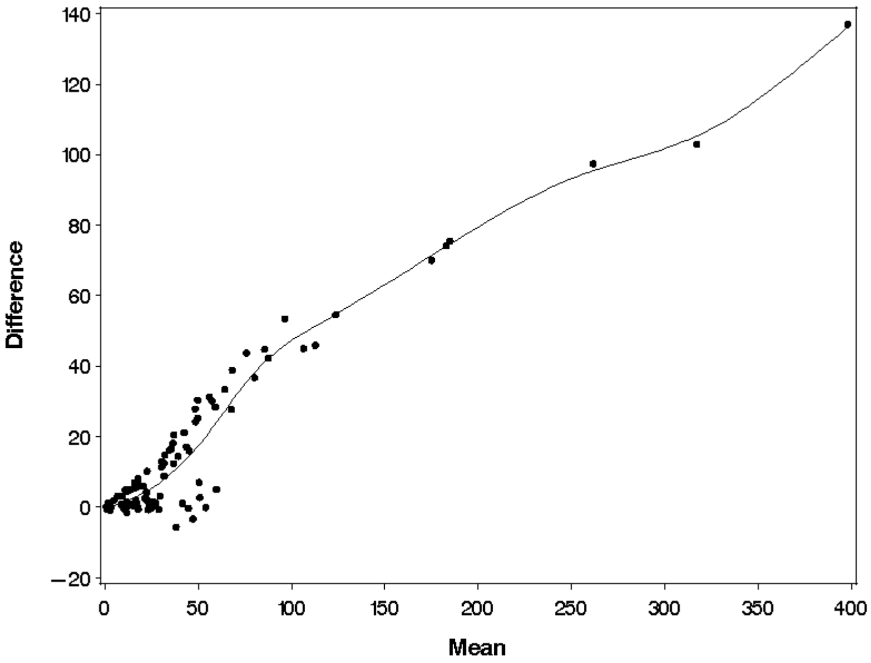
140
120
100
80
60
40
20
0
−20
Difference
0
50
100
150
200
250
300
350
400
Mean
